# The role of statins in both cognitive impairment and protection against dementia: a tale of two mechanisms

**DOI:** 10.1186/s40035-018-0110-3

**Published:** 2018-02-27

**Authors:** Bob G. Schultz, Denise K. Patten, Daniel J. Berlau

**Affiliations:** Regis University School of Pharmacy, 3333 Regis Boulevard H-28, Denver, CO 80221 USA

**Keywords:** Statin, Cognition, Dementia, Memory, Cholesterol, Alzheimer’s disease, Vascular dementia, Neuroprotection

## Abstract

Nearly 30% of adults 40 years and older in the United States are on a statin. Their widespread use heightens the importance of careful consideration of their varied effects on the body. Although randomized controlled trials have not confirmed cognitive impairing effects with statins, continuing evidence suggests statins have the ability to cause reversible cognitive impairment in some patients. Paradoxically, statins have also been shown to decrease the risk of dementia, Alzheimer’s disease, and improve cognitive impairment in some cases. However, randomized controlled trials have similarly failed to find the beneficial effect. Supporting evidence for both claims is compelling whereas known limitations of the clinical trials may explain the lack of findings. This narrative review aims to explain why there is still controversy and how both effects can, and may, be possible. The mechanisms that have been hypothesized for each effect are seemingly independent from one another and may explain the contradicting results. Being mindful of the complex effects of statins, health care providers need to be able to identify patients who are at risk for or already experiencing cognitive impairment from statin use while also identifying those who could potentially decrease their risk of dementia with statins.

## Background

Cardiovascular disease (CVD) is the leading cause of morbidity and mortality in the United States and elevated cholesterol is a major risk factor [1]. Statins are the most effective low density lipoprotein-cholesterol (LDL-C) lowering medications available and have been strongly associated with a reduced risk of atherosclerotic CVD [2]. In addition to their LDL-C lowering effects, statins also increase high density lipoprotein-cholesterol (HDL-C), decrease triglycerides (TG), and have numerous positive effects on the body [3]. As such, statins are currently the first line pharmacological therapy for the treatment of hyperlipidemia in the primary prevention of coronary heart disease (CHD). Statins have become some of the most prescribed drugs because of their pivotal role in decreasing the risk of CHD events and ischemic stroke with atorvastatin being the second most dispensed drug in the United States [4]. Over 32 million people in the United States (10% of Americans) are currently on a statin with an estimated 56 million people (24% of Americans) eligible to consider a statin. Fourty-1 % of patients on statins are 75 years and older, 42 % are between 65 and 74 years, and 17 % are between 45 and 64 years [5]. The widespread use of statins heightens the importance of careful consideration of their effects on the body.

Post marketing reports of statins have implicated a reversible cognitive impairing effect in some patients. In contrast, Phase III clinical trials used for drug approval did not report any significant increase in cognitive impairment in statin users versus placebo [6]. However, the clinical trials were not originally designed to detect cognitive impairment. Re-evaluations of the statin clinical trial data have also found no effect on cognition but case-reports and studies have continued to suggest statins can cause cognitive impairment in some patients. Moreover, the Food and Drug Administration (FDA) addressed the potential cognitive impairing effects in 2012 and recognized statins may cause reversible cognitive impairment in an announcement in 2012 [7]. Careful analysis of the current evidence is imperative to fully evaluate the state of knowledge on the effects of statins on cognition.

Statins have also, paradoxically, been associated with a reduced risk of dementia and slowed progression of AD. Observational studies have provided substantial evidence supporting a protective effect against the onset of dementia but are subject to a number of potential biases, such as selection bias and information bias [8]. Randomized controlled trials evaluating the effects of statins on cognitive measures in patients with AD have generally failed to show positive benefits but may have limited ability to detect therapeutic benefit by excluding subjects with dementia or dyslipidemia.

Statin clinical trials [9–11] and subsequent reevaluation of the clinical trial data [12] found no decreased risk of dementia. However, because these studies were specifically designed to evaluate CVD outcomes and not cognitive outcomes, it is possible that their design might limit their ability to detect effects on the risk of dementia.

The objective of this commentary is to review the current evidence of the claims on statin effects on cognition and discuss some potential mechanisms that would allow statins to lead to both cognitive impairment as well as a reduced dementia risk. Recognition of both potential mechanisms could help patients maximize the beneficial effects and avoid the potentially negative effects on cognition.

### Statins and reversible cognitive impairment – evidence for

Statins have been linked to short-term reversible cognitive impairment since early safety and tolerability testing [13]. During a phase I clinical trial of atorvastatin, escalating doses showed a dose-dependent adverse reaction of mild, transient, restlessness, euphoria, and mental confusion [13]. However, cognitive impairment was not reported significantly enough in the in phase II and III statin clinical trials to be recognized as a side effect [9–11]. Post-marketing surveillance can uncover side effects not identified in clinical trials and post-marketing surveillance of statins has revealed numerous case reports that characterize a transient and reversible cognitive loss. A review of 60 case reports described the main symptom of short-term memory loss that occurred a few months after the start of statin therapy or after an increase in dosage. Several case reports observed resolution of cognitive impairment upon discontinuation of the statin and recurrence of cognitive impairment upon rechallenging of the statin [14].

A double blind placebo-controlled trial of lovastatin’s effect on cognitive functioning and mood showed detrimental effects on cognitive performance on four neuropsychological tests assessing attention, working memory, and overall mental efficiency [15]. A follow up double blind, randomized trial found a significantly worsened learning effect in participants taking statins compared to placebo [16]. The participants on statins were not able to learn from prior experiences as well as those on placebo. The strength of evidence is heightened by the randomization and controlled structure in these studies however, the strength of evidence is lowered by the small population of only 308 participants.

A patient survey-based analysis found evidence of cognitive impairment with variable onset and recovery courses, a clear relation to statin potency, and a significant negative impact on quality-of-life [17]. The Naranjo adverse drug reaction probability scale [18] was used to describe the relationship between statins and cognitive impairment. The Naranjo score is widely recognized as an accepted tool to measure the correlation between an adverse event and a medication increasing the strength of this evidence. Seventy-five percent of the cognitive adverse drug reports were determined to be probably or definitely related to statin therapy. In some patients, a diagnosis of dementia or Alzheimer’s disease was reportedly reversed upon discontinuation of the statin. Patients whose symptoms improved after discontinuation reproduced the symptoms after the statin was rechallenged. Some of those patients were rechallenged multiple times with continued recurrence of the adverse effect. Quality-of-life measures were significantly reduced in these patients, establishing the seriousness of this potentially avoidable adverse effect [17].

Because of much of this evidence, as well as a thorough review of the Adverse Event Reporting System (AERS), the FDA announced a required label change informing patients and physicians that cognitive side effects are a risk when taking statins in 2012 [7]. The FDA described the symptoms as not serious and reversible with variability in symptom onset and resolution, but recognized the effects as potentially being caused by statin therapy. The FDA also stated that the cardiovascular benefits of statins outweigh these small increased risks. The small increased risks noted in the FDA’s announcement were determined by evaluating large clinical trials such as JUPITER which showed a trend towards an increased risk of reversible cognitive impairment defined by a relative risk of 1.33.

The evidence supporting reversible cognitive impairment has revealed sub-populations more likely to experience the side effect. Following the FDA’s announcement, researchers further examined the AERS database to explore the effects and if the evidence would allow for patients at higher risk to be identified [19]. The researchers identified 2597 reports of cognitive impairment and a significantly higher proportion of adverse reports of cognitive dysfunction associated with potent, lipophilic statins, specifically, atorvastatin and simvastatin, compared to less lipophilic statins and selected drugs commonly used in the general population. Although high-doses and lipophilicity may play a significant role in the incidence of reversible cognitive impairment, other factors such as race/ethnicity, gender, statin molecule, and genotypes may also play significant roles as well. Evidence has been mounting to identify other patient specific factors that may play a role in identification of patients at risk.

Voluntary reporting to AERs captures only an estimated 1 to 10% of true events. By extrapolation from their data the authors estimated a true amount of 3000 to 30,000 occurrences of cognitive impairment associated with atorvastatin and simvastatin a year [19]. Since there are 32 million Americans on statins the risk of cognitive impairment can be extrapolated to be between 0.1–1% for the entire population taking a statin.

Post-marketing surveillance has continued to uncover case reports of cognitive effects associated with statin use since the FDA report. A case series aimed to describe the potential behavioral and psychiatric changes in statin users and found most of the adverse reports met presumptive criteria for “probable” or “definite” causality by the Naranjo score [20]. Consistent with previous reports, lipophilic statins (atorvastatin, simvastatin, and lovastatin) were identified as the most commonly associated with cognitive impairment **(**Table 1**).**

**Table 1:** Evidence Supporting Cognitive Impairment

Study	Study Type	Statin	Participants	Findings/Relevance
FDA, 2012, [6]	Safety Literature Review	All	N/A	Statin labels should include cognitive impairment however the cardiovascular risks outweigh the small cognitive impairment risk.
Posvar EL, et al., 1996, [12]	Rising single-dose, partially blinded, three period study	Ator	22	Tolerance study that resulted in one participant experiencing cognitive side effects at the 120-mg solution dose. The participant experienced mild, transient restlessness, euphoria, and mental confusion that were considered to be dose-limiting side effects.
Wagstaff LR, et al., 2003, [13]	Review of case reports	Ator(23), Prava(1), Sim (36)	60	Case reports raise the possibility that statins may be associated with cognitive impairment in rare cases.
Muldoon MF, et al., 2000, [14]	Double-blind investigational	lova	209	Lovastatin treatment resulted in small performance decrements on neuropsychological tests of attention and psychomotor speed, the clinical importance of which is uncertain.
Muldoon MF, et al., 2004, [15]	Randomized trial	Sim	308	Patients given placebos performance improved more than statin patients on cognition tests given at day 0 and 6 months.
Evans MA, et al., 2009, [16]	Patient survey-based analysis	All	171	There is a characterizable association between statins and cognitive impairment. 128 patients experienced cogitive adrs determined to be probably or definitely related to statin therapy. Of 143 patients who reported stopping statin therapy, 128 reported improvement in cognitive problems, sometimes within days of statin discontinuation.
M Sahebzamani, 2014, [18]	FDA AERs database analysis	All	4867	Lipophilic statins (especially atorvastatin and simvastatin) have significantly more reports of Cog. Dys. than hydrophilic statins. Estimated Cog Dys reporting with atorvastatin and simvastatin is truly 3,00–30,000 reports/year.

*ator* atorvastatin, *flu* fluvastatin, *lova* lovastatin, *prav* pravastatin, *rosu* rosuvastatin, *sim* simvastatin

A recent case report describes two patients taking simvastatin who showed minor decreased cognitive function on neuropsychological tests but subjectively reported major differences in day-to-day functioning. The negative effects resolved upon discontinuation of simvastatin. Interestingly, the patients in the case report had controlled psychiatric conditions similar to previous case reports [20]. Patients with underlying behavioral conditions, not related to cognition, may be more vulnerable to the cognitive impairing effects [21]. The evidence seems to support a direct relationship between starting/rechallenging a statin medication and the development of cognitive impairment however, there is significant evidence against these claims.

### Statins and reversible cognitive impairment – evidence against

The FDA announcement drew controversy because previous to the announcement, the evidence supporting cognitive impairment was widely considered unconvincing [6]. The methods and detailed findings of the FDA’s announcement were not made available after the posting, causing confusion about their supporting evidence. A systematic review by Richardson et al. aimed to address the lack of RCT data supporting the FDA announcement [22]. The authors were unable to find any evidence of cognitive impairment associated with statins and found the AERs reporting rates of cognitive impairment to be similar between statins and other commonly used medications [22]. The authors concluded data do not suggest an effect of statins on cognition although they admitted the strength of the available data is limited, particularly with regard to high-dose statins.

Previous work had aimed to assess cognitive function in community-dwelling elderly participants treated with statins compared with placebo. The population-based sample underwent neurocognitive tests throughout the study including global cognitive performance, frontal-executive function, verbal fluency, and memory. Participants taking statins did not differ from their untreated counterparts in the performance of formal neuropsychological tests [23]. There still remained some questions regarding how usage consistency may play a role in the cognitive effects. A retrospective cohort study attempted to uncover the difference in psychological and cognitive disorders between persistent and non-persistent statin users. After analyzing statin users and ICD-9 codes for psychological diseases the outcomes data from the study showed that non-persistent statin users actually had a greater risk of being diagnosed with cognitive disorders compared with persistent users [24]. The authors hypothesized persistent statin use may be protective against developing cognitive dysfunction. However, the findings may be a result of reverse causality since non-persistent patients may be non-persistent because of their cognitive condition.

A systematic review and meta-analysis of randomized controlled trials (RCTs) was designed to address the FDA announcement regarding the effect of statins on cognition as well [6]. Cognitive impairment was rarely reported in trials involving either cognitively normal or impaired participants. Meta-analyses also showed no difference in the incidence of mild cognitive impairment between statin users and non-users. Additionally, the review found no significant effects or modification of effects across all neurocognitive domains, regardless of whether the drug penetrates the blood-brain barrier or not, study duration, sample size, location, or cognitive health status. The authors concluded that the FDA should re-evaluate their announcement about potential adverse effects of statins on cognition. An additional systematic review and meta-analysis [25] evaluated the effects of statins on short-term cognitive function in cognitively normal adults. The authors concluded that the available RCTs suggested no effects of statins on short-term cognition [25].

The 2013 The American College of Cardiology/American Heart Association (ACC/AHA) guidelines reviewed the RCTs assessing safety and efficacy and did not find evidence that statins had an adverse effect on cognitive changes or the risk of dementia [26]. The guidelines recommend avoiding consideration of cognitive impairment as a symptom into when prescribing statins. A summary of the papers suggesting no cognitive impairment from statin can be found in Table 2.

**Table 2:** Evidence Opposing Cognitive Impairment

Study	Study Design	Statin	Participants	Findings/Relevance
Ott BR, et al., 2015, [5]	Systematic review and meta-analysis	All	56,655	Statin therapy was not associated with cognitive impairment in RCTs.
Richardson K, et al., 2013, [21]	Systematic review	All	56,043	Larger and better-designed studies are needed to draw unequivocal conclusions about the effect of statins on cognition. Published data do not suggest an adverse effect of statins on cognition; however, the strength of available evidence is limited, particularly with regard to high-dose statins.
Benito-León J, et al., 2010, [22]	Population based study	Ator, flu, lova, prav, sim,	548	Statin users and controls performed similarly on neuropsychological tests.
Lilly SM, et al., 2014, [23]	Retrospective database investigational study	Ator, flu, lova, prava, rosu, sim	13,626	Non-persistent statin users had a greater risk of being diagnosed with schizophrenia/psychosis and cognitive disorders compared with persistent users.
Swiger KJ, et al., 2013, [24]	Systematic review and meta-analysis	All	23,541	In patients without baseline cognitive dysfunction, the results of the available studies are most compatible with no significant short-term cognitive detriments related to statin therapy, whereas long-term data suggest a beneficial role in the prevention of dementia.
McGuiness B, et al., 2016, [11]	Systematic review	Prava, Sim	26,340	There were no differences between statin and placebo groups on five different cognitive tests.

*ator* atorvastatin, *flu* fluvastatin, *lova* lovastatin, *prav* pravastatin, *rosu* rosuvastatin, *sim* simvastatin

### Study limitations

Even though substantial research has found no cognitive impairment with statin use there are limitations that may make drawing precise conclusions more difficult. The Justification for the Use of Statins in Primary Prevention (JUPITER) trial, Heart Protection Study (HPS), and Pravastatin in Elderly Individuals at Risk of Vascular Disease (PROSPER) are the primary RCTs that were unable to find increased cognitive impairment with statin use. However, the strength of the findings are poor in the RCTs because cognitive impairment was not a primary or even secondary outcome. Subsequently, the JUPITER Trial did not employ objective cognitive assessments to detect impairment; only incidence of dementia and self-reported serious side effects were tracked. Out of 17,802 participants in the JUPITER trial, 12 and 9 patients developed dementia in the rosuvastatin arm and placebo arm respectively. Additionally, 18 vs 4 patients reported confusion in the rosuvastatin and placebo arm respectively. Although the reports of confusion were not significantly increased in the rosuvastatin group there was a trend towards an increase in reported confusion. Also, self-reports are likely to be underreported because patients who do not have a noticeable limitation of activity or patients who fear being stigmatized for having cognitive dysfunction may not report [27].

HPS did evaluate cognition, however not at baseline, only upon the last follow up. Out of 20,536 participants 24% were classified as cognitively impaired by telephone-administered cognitive assessment at the end of the study in each treatment arm. The 24% cognitive impairment finding in each group is unexpectedly high and may suggest the tool used to screen for cognitive impairment may not have been sensitive. According to a review looking at validated instruments for assessment of cognitive impairment, telephone-based screening tools are useful in clinical based practice but need refinement in the validation steps to improve specificity and sensitivity. PROSPER, assessed the effects of pravastatin on cognition throughout the study however, pravastatin has not been shown to be able to cross the blood brain barrier [28] and the findings should not be extrapolated to other statins that do cross the blood brain barrier.

In addition to previously listed limitations, the statin clinical trials required strict inclusion/exclusion criteria. The HPS trial used an active drug compliance run-in, with the combined run-ins leading to 33% of patients being excluded. All patients were given 4–6 weeks of simvastatin prior to randomization. Patients were excluded if they were not compliant and could also be excluded if the general practitioner considered there to be a clear contraindication. Non-compliance could be a result of cognitive impairment and non-persistent users have been shown to have a higher incidence of cognitive impairment [24]. It’s possible that by excluding patients who were non-adherent or experiencing unacceptable side effects the investigators also excluded patients who were experiencing cognitive effects.

As stated above, patients with a medical history of psychiatric problems and alcohol abuse may be the most susceptible subset of patients to cognitive impairment from statins. Many of the case reports of cognitive impairment with statins were reported in those with bipolar disorder, schizophrenia, and other behavioral disorders [14, 20, 21]. RCTs that found no increased incidence of cognitive impairment often excluded those with a history of psychiatric problems and alcohol abuse [29]. A six-month double blind RCT measuring the change from baseline for four primary cognitive indices within treatment arms of low dose simvastatin, low dose pravastatin, and placebo included participants with preexisting psychiatric conditions failed to find significance but showed consistent trends toward cognitive impairment in the subset of patients with psychiatric conditions. Thus, many studies may be excluding subsets of patients that are more likely to have cognitive impairing effects, particularly those with psychiatric conditions.

As higher statin doses are more likely to cause cognitive impairment [13], the fact that only 20% of the RCTs reviewed employed doses at the upper limit of current guidelines could have affected their ability to detect effects from high dosages [6]. Real world usage of statins likely results in higher doses of statins due to lack of response to low doses [30]. Therefore, the low incidence of cognitive impairment reported in these RCTs may not accurately predict the incidence at higher statin doses which is a sub-population of patients at higher risk for reversible cognitive impairment. Additionally, interacting medications or patient comorbidities such as liver disease can lead to an increased exposure to statins. These real world factors were controlled in some RCTs examining statins and may have prevented detection of sub-populations at risk for experiencing cognitive impairment by excluding patients at a higher exposure to statins because of decreased metabolism and excretion.

Some researchers support the evidence for cognitive impairment but propose there is a biologic plausibility for a positive cognitive effect in some statin users as well [17]. Recognition of cognitive impairment as a side effect of statins does not mean there is a net cognitive impairing effect of statins. The beneficial effects of statins on prevention of ischemic stroke may improve cognition by decreasing severity of cerebrovascular disease [31]. The potential beneficial effect of statins on cognition may negate the reports of cognitive impairment in previous randomized controlled clinical trials, explaining why there was no cognitive impairing effect found.

### Statin benefits in preventing and treating dementia and Alzheimer’s – evidence for

Statins for the prevention and treatment of dementia began generating attention when two epidemiologic studies reported a lower risk of dementia in statin users [32, 33]. Since then, many medical professionals have investigated the potential beneficial effects of statins on long-term cognition and decreased incidence of cognitive related disease.

The protective effects of statins have been examined in a variety of reviews and meta-analysis [34]. A meta-analysis that included observational studies and a randomized control study found a significant protective effect against all-cause dementia and Alzheimer’s disease with statin users [35]. When compared to non-statin users, those who used statins had a significantly lower relative risk (0.82) of developing all-type dementia [35].

An updated review of statins and cognition analyzed statin’s effect on slowing the progression of dementia and AD and in preventing the differing types of cognitive impairment, including dementia, Alzheimer’s disease, and cognitive impairment [36]. The review investigated numerous studies including two RCTs studying the treatment of AD with atorvastatin and simvastatin that found no significant benefit on cognition, measured by ADAS-Cog, or global functioning, measured by Alzheimer’s Disease Cooperative Study Clinical Global Impression of Change (ADCS-CGIC), over 72 weeks in patients with mild-moderate AD. However, these RCTs did not include patients with increased lipid levels which may be a subpopulation that would show the most benefit from a cholesterol-lowering statin. Also, the short duration and inclusion of patients who were already having clinical signs of AD may impede the strength of the evidence. Another RCT showed a decrease in 24S–hydroxycholesterol in the CSF of patients with mild AD [37]. A reduction in 24S–hydroxycholesterol is correlated with a reduction in amyloid beta-40 (Aβ40) giving rise to a potential treatment for AD. However, no improvements in MMSE or ADAS-Cog were found in any patients, including the patients with reduced 24S–hydroxycholesterol. The effect of simvastatin and atorvastatin on the prevention of incident dementia was illustrated in a large retrospective cohort study. The authors found a significant decrease in the incidence of incident dementia in the atorvastatin group. Most of the data reporting a protective effect of statins on dementia and AD in patients with normal cognition are observational leading the authors of the review to conclude that additional RCTs are needed to develop a causal relationship, but the data showed a decreased risk of dementia in statin users.

The results from two studies found that the benefits were confined to persons under the age of 80 years which may suggest statins may lose their beneficial effects for cognition at a certain point in life [38, 39]. The adjusted hazard ratio for those under the age of 80 was significantly lower (0.44) while the adjusted hazard ratio for persons over the age of 80 was 1.22. The Mirage study showed a significant decrease in the risk of Alzheimer’s disease among statin users even after adjusting for race and APOE genotype [40]. Race and APOE genotype have been shown by numerous studies to play a role in both the development of AD and the effectiveness of statins in the treatment of AD. A retrospective study found a lower risk of AD in white women, white men, Hispanic women, Hispanic men, and black women taking simvastatin [41]. Additionally, a lower risk of AD was found in white women, black women, women overall, and Hispanic men taking atorvastatin [42]. Another retrospective study looking at failed clinical trials found that simvastatin use may slow the progression of cognitive decline and to a potentially greater therapeutic efficacy in those homozygous for APOE4 [43]. Mounting evidence supports varied therapeutic benefit of statins across different statin molecules, sex, race/ethnicity, and genotyping leading to the need for more studies that aim to develop statin precision-medicine and stratification of patient populations.

Statin use was associated with significantly reduced risk of incident Alzheimer’s disease after adjustment for age, gender, education, and APOE genotype. A significant, but lesser, protective effect was found with non-statin lipid lowering agents conferring that the protective effect from statins may be due to their lipid lowering effects instead of potentially other novel mechanisms [44]. However, most studies in the literature have found no association between non-statin lipid-lowering drugs and the risk of dementia [45].

Because statins intended effect on the body is lowering cholesterol, many researchers have attempted to associate cholesterol levels with the adverse or positive effects of statins. High total cholesterol (TC) in midlife was associated with significant increased risk of developing late-life Alzheimer’s disease compared to normal cholesterol [46]. However, high TC in late-life was not associated with mild cognitive impairment (MCI), Alzheimer’s disease, vascular dementia, any dementia, or cognitive decline [46]. Although statins were not examined, the results suggested lower cholesterol levels in midlife with a statin may decrease the risk of Alzheimer’s disease in late-life. This effect was confirmed by a recent study that found elevated TC and TG were associated with a greater decline on a test of executive function, sustained attention, processing speed, memory scores, and a summary measure of cognition [47]. Also, results from a re-analysis of a simvastatin RCT were consistent with previous work as no association between elevated TC or TG and Alzheimer’s disease in late-life was found from a combined set of AD trials and in observational studies [43]. Additionally, a higher baseline LDL and TC has been associated with increased risk of developing Alzheimer’s disease, whereas higher baseline TG concentrations were associated with vascular or mixed dementia [48]. There is also evidence of a decreased rate of functional decline in statin users with incident Alzheimer’s disease. Statin use, along with use of beta-blockers, was found to be associated with delay of functional decline via The Clinical Dementia Rating-Sum of Boxes [49] after a mean of three years of follow up. However, further studies are needed to confirm the findings.

A recent comparative effectiveness study investigated whether fungus-derived statins are associated with an lower risk of incident AD compared with synthetic statins [50]. The researchers used real-world clinical practice data and found that fungus-derived and lipophilic statins were not associated with decreased incidence of AD compared to synthetic and hydrophilic statins. Interestingly, the study found fungus-derived statins and lipophilic statins were associated with higher AD risk compared to synthetic and hydrophilic statins respectively. Although more research needs to be done comparing the heterogeneous nature of statin effects on incident AD based on their characteristics, statins cannot be presumed to have a beneficial or detrimental effect compared to non-statin users from this study.

### Statin benefits in preventing and treating dementia and Alzheimer’s – evidence against

The role of statins in the prevention of dementia was not found to be beneficial as a Cochrane Systematic Review did not find any protective effects of statins [12]. The review examined two of the primary RCTs previously mentioned, HPS and PROSPER, which together included 26,340 participants [11, 51]. The HPS study reported no difference in the incidence of dementia between the two groups. The PROSPER trial also showed no difference from statin use on any of their cognitive measurements. Similarly, the researchers reported no evidence that lipid-lowering therapy, particularly with statins, prevented vascular dementia or slowed the progression of Alzheimer’s disease late in life [52].

As mentioned above, there is reason to believe, however, that these large clinical trials may not have been ideally designed to detect the effects that statin had on the risk of dementia and cognitive decline. The PROSPER trial did not measure incidence of dementia directly and may have included participants too old to see a benefit on risk of Alzheimer’s disease or dementia from statin therapy by only including patients 70–82 years old. Evidence suggests that lowering cholesterol in midlife, but not late life, is associated with lower risk of dementia [47], and therefore these participants may have received statin therapy too late in life to see a benefit. The HPS study did measure incidence of dementia as an outcome but it was unclear from the study results which criteria were used to diagnose dementia [12].

### How could statins do both?

Considering the evidence presented above, it is conceivable that statins can contribute to both reversible cognitive impairment as well as preservation of cognition through the prevention of dementia. If statins have both of these effects, there are a variety of possible explanations as to how this is occurring. The various physiological effects statins have on the body can explain how they can cause a myriad of different outcomes.

### Statins and reversible cognitive impairment – proposed mechanisms

Cholesterol is vital to normal brain functions including learning and memory [53]. It is important in myelin sheath formation, mitochondrial function, neurotransmitter receptor expression, synapse development, production of steroid hormones involved in brain and peripheral signaling, and the transport of antioxidants such as coenzyme Q10 (Co Q10) [17]. It is unlikely that decreasing peripheral serum cholesterol levels affect cognition, however, it is more probable that affecting cholesterol levels locally in the CNS is responsible for the cognitive impairing effects [53]. Increased exposure to a medication (e.g. high doses) and increased lipophilicity can result in increased brain exposure to a medication [54] leading to the hypotheses that higher statin potency/doses and lipophilicity may translate to decreased cholesterol levels locally in the brain and subsequently lead to cognitive impairment.

Increased exposure to statin medication has been shown to cause cognitive side effects. High doses and poor metabolism can lead to increased exposure to medications. The dose dependent cognitive impairment relationship reported in the atorvastatin safety study [13] suggests that high doses cause cognitive side effects. By exposing the body to more drug, the concentration gradient begins to favor diffusion into the CNS [55], allowing statins to potentially effect cholesterol synthesis locally in the brain. Individual genetic factors, such as genes encoding for cytochrome P450 enzymes, mitochondrial enzymes, influx transporters, and efflux transporters, can alter the body’s exposure to statins as well [56] making the case that some patients may be more vulnerable to cognitive impairment due to their genetics.

However, lower intensity, lipophilic statins (i.e. lovastatin) have reported cognitive impairment effects as well. Lipophilic statins cross more readily into the central nervous system in animal and in vitro studies [28]. Increased permeability into the blood brain barrier translates to decreased cholesterol levels locally in the CNS [53], potentially lowering cholesterol below the level required for normal cognitive functioning. This may suggest that lipophilicity coupled with over-exposure may be responsible for the incidence of cognitive impairment. Lipophilic statins also passively and non-selectively diffuse into both hepatocyte and non-hepatocyte tissue while hydrophilic statins rely largely on active transport into hepatocyte to exert their effects [57–61]. High selectivity for hepatocytes is thought to translate into a reduced risk for adverse effects [38, 58]. Statins that have low selectivity for hepatocytes tend to achieve higher levels of exposure in non-hepatic tissues, including the brain. Of the statin related cognitive impairment reports, the vast majority are simvastatin or atorvastatin [19]. More widespread use of atorvastatin and simvastatin versus other statins result in higher reports of adverse effects. However, atorvastatin and simvastatin have a higher proportion of cognitive impairment reports compared to other statins as well suggesting their lipophilicity may be responsible for the increased incidence of cognitive impairment.

Low cholesterol in neuronal membranes may lead to lower lipid microviscosity which could affect neurotransmitter synthesis resulting in decreased synaptic binding and uptake [62]. Recent animal and in vitro studies suggest that alteration in cholesterol synthesis and degradation profoundly influences higher-order brain function [63]. Additionally, animal and in vitro studies have shown cholesterol is critical for myelin sheath formation and decreasing cholesterol levels in the CNS could negatively impact the formation of the myelin sheath and affect information processing and cognition [53]. Because cholesterol levels return to baseline after discontinuation of the statin, this mechanism could explain the reversible characteristic of the cognitive impairment.

Statins could also be causing cognitive impairment by interfering with mitochondrial function in the brain. Patients with biologic mitochondrial or metabolic vulnerability such as metabolic syndrome factors, thyroid disease, and genetic mutations linked to mitochondrial dysfunction have proven to be risk factors for developing adverse effects from statins [64]. Although these factors primarily affect muscle function, the brain is also vulnerable to mitochondrial defects similar to muscle tissue [65]. Statins also have a dose dependent effect on the lowering Co Q10 levels which can affect mitochondrial function by playing an important role in energy production and free radical defense throughout the body. Therefore, lowering Co Q10 and cholesterol locally in the CNS could be responsible for the cognitive impairing effects seen in some patients, which may be a different mechanism from how statins can reduce risk of dementia.

### Statins in prevention and treatment of dementia and Alzheimer’s disease – proposed mechanisms

Most of the proposed mechanisms relate to the effects of statins on the cerebrovascular system [66]. Dementia has been proposed to be caused by large vessel disease and/or microvascular damages and risk factors for each are slightly different. Statins may be able to show benefits in each pathology. The most evident protective effect statins have on cognition is the prevention of stroke and possible subsequent vascular dementia. The effects of statins may not only be affecting stroke risk, but may also be preventing microvascular infarcts that lead to dementia without an acute stroke.

Statins have been linked with reduced risk of all-cause dementia and even Alzheimer’s disease as well. It is possible that statins are affecting Alzheimer’s pathology directly [67]. Polymorphisms in the 3-hydroxy-3-methyl-glutaryl-coenzyme A (HMG-CoA) reductase gene are genetic modifiers for the risk, age of onset, and conversion of Alzheimer’s disease [68]. Studies have also shown a greater benefit of statins on decreased risk of developing dementia or Alzheimer’s in patients who have congenital abnormalities subjecting them to hyperlipidemia [45]. This could suggest that the positive effect of statins on long term cognition is by restoration of cholesterol homeostasis in those who have congenital hyperlipidemia conditions. An optimal local cholesterol level may be necessary for normal physiologic processing. Increased amounts of local cholesterol may result in the formation of Aβ while decreased amounts may prevent normal physiologic functioning and cause the reversible cognitive impairment.

Additionally, statins could reduce dementia risk by directly affecting Alzheimer’s disease pathology. A study in transgenic mice models of Alzheimer’s disease found that atorvastatin reduced Aβ formation [69], and atorvastatin can attenuate some the damage from neuroinflammation in Alzheimer’s disease [70]. In order for atorvastatin to be able to effect Aβ formation in the brain and attenuate damage from neuroinflammation it must be able to be lipophilic to cross the blood brain barrier. Statins have also been hypothesized to have potentially positive effects on isoprenoids and the consequences of protein prenylation in the brain. Isoprenoids, particularly farsenylpyrophosphate (FPP) and geranylgeranyl pyrophosphate (GGPP), have recently been shown to be elevated in human AD brain tissue [71] and statins have been shown to reduce the amount of mevalonate, a precursor to FPP and GGPP. However, evidence of FPP and GGPP regulation in the brain is limited and even more so in regard to statins’ effects on the isoprenoids in the brain [72]. Conversely, it is also possible that statins are affecting risk of cerebrovascular associated dementia and this dementia is being called all-cause or even misdiagnosed at Alzheimer’s disease. Vascular dementia in the absence of an acute stroke and Alzheimer’s have similar clinical presentations and vascular dementia sometimes goes undetected on imaging and is diagnosed as Alzheimer’s disease [73]. Therefore, statin effects on Alzheimer’s disease in the literature may actually be an effect on this gradual onset vascular dementia.

The protective effects of statin may also be independent of their cholesterol lowering effects entirely. This is supported by evidence of statins’ ability to reduce ischemia in the brain by affecting endothelial cell function and blood flow, decreasing LDL oxidation, enhancing the stability of atherosclerotic plaques, inhibiting vascular smooth muscle proliferation and platelet aggregation, and reducing vascular inflammation [3]. Additionally, statins inhibit Rho GTPases in vascular wall cells leading to increased expression of atheroprotective genes and inhibition of vascular smooth muscle cell proliferation [74].

## Conclusion

The current literature supports the hypothesis that statins are potentially responsible for both reversible short-term cognitive impairment as well as a decreased risk of dementia. The strength of evidence is insufficient and has significant limitations unique to each study design however there is growing evidence for reversible cognitive impairment for a small percentage of the population of statin users and strengthening evidence for statins reducing risk of AD and dementia. Additional RCTs will be required to conclusively determine the global effects of statins on the body. Since RCTs have their own limitations, strict inclusion/exclusion criteria, studies that utilize data from real-world evidence will be increasingly important. The recently-passed twenty-first Century Cures Act may provide credibility to population based studies which would allow important research to be conducted on this topic using large data sets.

The hypothesized mechanisms for each of these effects are likely independent from each other and can occur simultaneously. Not all patients may be affected by these medications in the same way and therefore work has begun identifying subsets of patients that may have be more especially susceptible to one or both of these effects [56]. Potential users of statins should be aware of these possible effects in order to better identify the effects if they occur. The 2013 ACC/AHA treatment of blood cholesterol guidelines do agree, however, that this potential reversible cognitive impairment should not be a reason to avoid statin use [26].

Health care providers need to be able to identify patients who are at risk for or already experiencing cognitive impairment from statin use while also identifying those who could potentially decrease their risk of dementia with statins. Patients who have genetic or biologic factors (e.g. decreased drug metabolism) that would predispose them to increased exposure to statins are indicatively at a higher risk for adverse effects, including cognitive impairment. Additional to identification of patients at risk of increased exposure, lipophilicity of the statin should also be considered. The patients at highest risk of cognitive impairment are those that are at risk of increased exposure, those with metabolic syndromes, and who are taking lipophilic statins. Much of the evidence supporting statins in the prevention of dementia and AD are in persons exposed to statins at mid-life as opposed to late life. This suggests that statins benefits may be limited to the vascular prevention stage of AD and dementia. Limited evidence suggests the decreased risk of dementia seen with statins may be limited to patients with hyperlipidemia in mid-life. By understanding the full spectrum of statin effects, health care providers can maximize the amount of patients that receive the potential benefits on long-term cognition and minimize those who might be affected by cognitive impairment.

## Abbreviations

ACC: American College of Cardiology
AERS: Adverse Event Reporting System
AHA: American Heart Association
CHD: coronary heart disease
Co Q10: Coenzyme Q10
CVD: Cardiovascular Disease
FDA: The Food and Drug Administration
HDL-C: high density lipoprotein-cholesterol
HMG-CoA: 3-hydroxy-3-methyl-glutaryl-coenzyme A
HPS: Heart protection study
JUPITER: Justification for the use of statins in primary prevention trial
LDL-C: lipoprotein-cholesterol
MCI: Mild cognitive impairment
PROSPER: Pravastatin in Elderly Individuals at Risk of Vascular Disease
RCTs: Randomized controlled trials
TC: Total cholesterol
TG: Triglycerides
